# Computational Mass Spectrometry–Based Proteomics

**DOI:** 10.1371/journal.pcbi.1002277

**Published:** 2011-12-01

**Authors:** Lukas Käll, Olga Vitek

**Affiliations:** 1 Science for Life Laboratory, Royal Institute of Technology, Stockholm, Sweden; 2 Department of Statistics, Department of Computer Science, Purdue University, West Lafayette, Indiana, United States of America; Whitehead Institute, United States of America

**This is an original** ***PLoS Computational Biology*** **tutorial.**

## Goals and Challenges of Proteomics

Proteomics is defined as the system-wide characterization of all the proteins in an organism in terms of their sequence, localization, abundance, post-translational modifications, and biomolecular interactions. Modern proteomic investigations are increasingly quantitative and comprehensive [1]. Examples include the relative quantification of over 4,000 proteins in haploid and diploid yeast, which identified the pheromone signaling pathway as enriched in differential abundance [2]; determination of site- and time-specific dynamics of more than 6,000 phosphorylation sites of HeLa cells stimulated with epidermal growth factor [3]; and characterization of 232 multiprotein complexes in *Saccharomyces cerevisiae*, which proposed new cellular roles for 344 proteins [4]. Such investigations are now successfully utilized in functional biology [5], [6], genomics [7], [8], and biomedical research [9].

**Figure 1:**
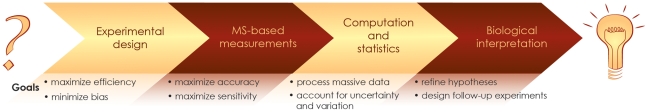
Quantitative mass spectrometry–based proteomic workflow. The workflow requires a tight integration of biological and experimental (red) and computational and statistical (yellow) analysis steps.

Challenges of proteomic studies stem from the complexity of the proteome and to its broad dynamic range. For example, the human genome contains around 20,000 protein coding genes. Their translation, combined with splicing or proteolysis, yields an estimated 50,000–500,000 proteins, and over 10 million different protein forms can be derived by somatic DNA rearrangements and post-translational modifications [10]. The abundance of protein species in human plasma spans more than 10 orders of magnitude [11]. Unlike oligonucleotides, proteins cannot be amplified, and therefore the objectives of proteomics are achieved by sensitive and scalable technologies identifying and quantifying proteins [12]. The overall mass spectrometry–based proteomic workflow is summarized in Figure 1.

## Experimental Design

**Figure 2:**
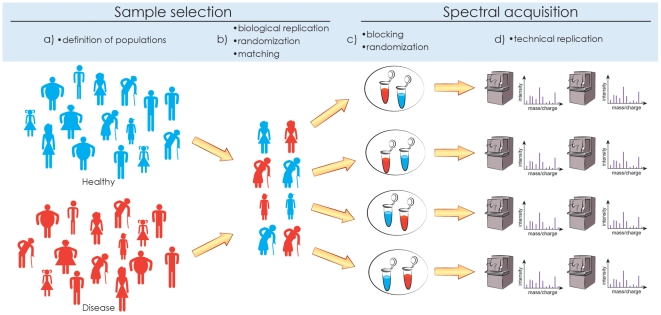
Experimental design. Statistical experimental design consists of (a) defining the populations of interest, (b) randomly selecting biological replicates from the population and (optionally) matching confounding factors, (c) randomly allocating biological samples to spectral acquisition and (optionally) grouping the samples in balanced blocks for joint profiling, and (d) (optionally) acquiring technical replicate measurements on the biological samples. Replication, randomization, and blocking are necessary to avoid biases and maximize the efficiency of the experiment.

Quantitative proteomic investigations are conducted in the context of biological variation [13], technical variation due to sample processing and spectral acquisition, and ambiguities of spectral interpretation. Statistical experimental design [14], [15] accounts for these sources of variation. The first goal of experimental design is to avoid biases [16], [17] (i.e., systematic errors in interpretation) by clearly defining the populations of interest, matching the individuals with respect to the confounding factors, randomizing the selection of matched individuals from the population, and randomizing sample allocation to the processing steps. The second goal is to ensure efficiency (i.e., minimal random variation and uncertainty for a given cost) by choosing an appropriate number of biological and technical replicates, and by allocating the replicates to experimental resources in balanced blocks. The steps of the statistical experimental design are summarized in Figure 2.

## Mass Spectrometry–Based Measurements

### Global Label-Free LC-MS/MS Workflow

**Figure 3:**
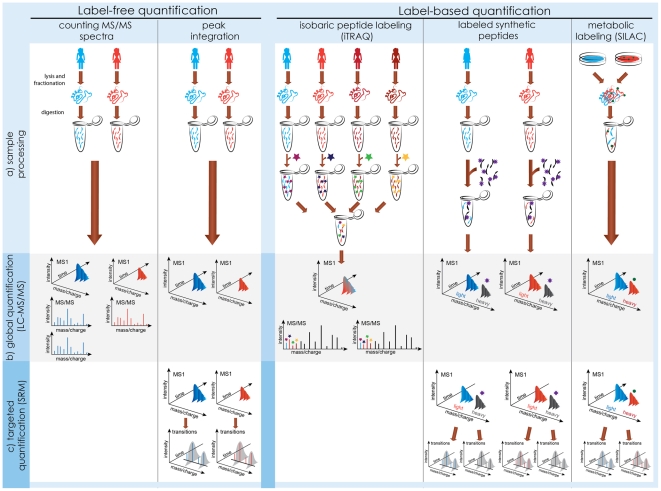
Mass spectrometry–based measurements. (a) Sample processing. Label-free quantification requires minimal sample manipulation, and acquires spectra from each sample in a separate mass spectrometry run. Label-based quantification varies in the timing and type of the labeling steps, but always simultaneously profiles two or more biological samples within a run. (b) Global label-free workflows achieve relative quantification by comparing counts of MS/MS spectra, or intensities of MS peaks between runs. Global label-based workflows compare intensities of reporter MS/MS fragments (iTRAQ) or MS peaks (SILAC, synthetic peptides). (c) Targeted workflows are an alternative to global quantification. They are most sensitive, but require an *a priori* knowledge of the proteins of interest, and of the technological characteristics of their peptides. Label-free targeted experiments compare intensities of transitions between runs, and label-based experiments within a run.

Mass spectrometry is currently the only technology for protein identification and quantification that is both high-accuracy and high-throughput [18]–[20]. Although many alternatives exist, shotgun liquid chromatography coupled with tandem mass spectrometry (LC-MS/MS; overview in Figure 3) is most frequently used. Mass spectrometry is better amenable to characterizing peptides; therefore, LC-MS/MS starts by enzymatically digesting proteins into a peptide mixture. Next, liquid chromatography (LC) separates the peptides, and the separated peptides are ionized and further separated by the mass spectrometer according to their mass-to-charge ratio in a mass spectrum (MS). The mass spectra obtained from the same sample at different elution times form an LC-MS run, and intensities of MS peaks, are related to peptide abundance. For identification, the mass spectrometer isolates the biological material of selected MS peaks, subjects it to collision energy or another type of fragmentation, and separates the resulting fragments in a secondary (MS/MS) mass spectrum. The distances between the MS/MS peaks are used to infer the amino acid sequence of the parent MS peak. Since abundant MS1 peaks are more likely to be selected for fragmentation, relative peptide quantification can also be achieved by counting the number of identified MS/MS spectra.

An LC-MS/MS experiment can identify and quantify thousands of proteins in complex mixtures. It requires minimal manipulation of the sample, and minimal prior information regarding its composition. However, the workflow has a number of deficiencies. Enzymatic digestion increases the complexity of the mixture. For example, a proteome comprising 5,000 proteins is expected to yield over 250,000 tryptic peptides, and minor cleavage and fragmentations of abundant proteins can obscure major events of low-abundant proteins, complicating the interpretation [21]. Dynamic range of mass spectrometers is limited to 3–4 orders of magnitude, and the direct LC-MS/MS analysis is biased towards most abundant peptides [22]. Technical variation can further undermine the identification and the quantification steps. A variety of extensions to this basic workflow have therefore been proposed.

### Overcoming Between-Run Variation: Label-Based Quantification

The LC-MS/MS workflow is enhanced by labeling samples from different conditions metabolically (e.g., with SILAC [23], where stable isotopes are included in the growth medium of an organism), or chemically (e.g., with iTRAQ [24] or TMT [25], where reacting chemical labels are applied during sample processing). Samples with different labels are combined and analyzed by a mass spectrometer within a single LC-MS run. Peaks from the samples are subsequently recognized by label-induced mass shifts in MS (SILAC) or MS/MS (iTRAQ, TMT) spectra, and used for relative quantification. Labeling enables within-run comparisons of protein abundance, and improves the precision of quantification. Experimental design can further gain efficiency through optimal allocation of samples to the labels, e.g., in reciprocal or reference designs [26] or by using labeled synthetic peptides as references. However, labeling requires extra sample manipulation and increases the complexity of the sample.

### Overcoming Limits of Dynamic Range: Targeted Workflows

The complexity of a biological mixture can be overcome by fractionation [27]; however, this severely undermines the throughput. A valuable alternative is selected reaction monitoring (SRM) (also referred to as multiple reaction monitoring, MRM), a targeted workflow where the mass spectrometer isolates a set of pre-defined peptides and their fragments during mass analysis [28]–[31]. The resulting peptide-fragment pairs (called transitions) are used for quantification. Since the isolation is highly specific, SRM enables the most sensitive mass spectrometry–based quantification currently available. For example, proteins expressed with fewer than 50 copies/cell were quantified in total yeast lysates [32]. As shown in Figure 3, SRM can be conducted in conjunction with both label-free and label-based workflows. The drawback of targeted workflows is that they only quantify *a priori* known proteins, require optimized experimental protocols, and limit the number of measurements per run to a few hundreds. Further technological developments [33] and optimal experimental designs [34] will help alleviate these drawbacks.

## Computation and Statistics

### Identification of Peptides and Proteins

**Figure 4:**
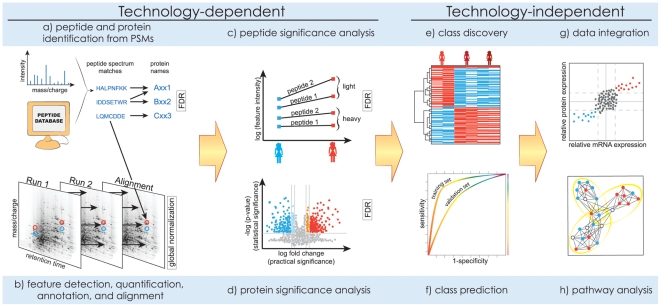
Computation and statistics. Analysis of the acquired spectra includes (a, b) signal processing, (c, d) significance analysis, and (e–h) downstream analysis. Methods in (a–d) must reflect the technological properties of the workflows. Methods in (e–h) are technology-independent and are similar to the analysis of gene expression microarrays, but their use is affected by uncertainty in protein identities and the incomplete sampling of the proteome.

The computational and statistical analyses of the acquired spectra are illustrated in Figure 4. With the shotgun LC-MS/MS workflow, the first step is to identify sequences of amino acids that correspond to the MS/MS spectra. This has received much attention from both algorithmic and statistical viewpoints [35]–37. A predominant approach is the database search, which compares each observed spectrum to the theoretical spectra predicted from a genomic sequence database (or to the previously identified experimental spectra in a library [38]), and reports the best-scoring peptide-spectrum match (PSM). Emerging alternatives are *de novo* identifications and hybrid searches [39], [40].

Due to the stochastic nature of the MS/MS spectra [41], and to deficiencies of scoring functions and databases, the best-scoring PSMs are not necessarily correct. Statistical characterization of the identifications is necessary, and is now required by most journals [42]. This problem is frequently formalized as controlling the false discovery rate (FDR) in the list of reported PSMs [43], [44]. Representative methods for controlling FDR are two-group models, which view the reported PSMs as a mixture of correct and incorrect identifications [45], and methods utilizing decoy databases [46]. Typically, only around 30% of MS/MS spectra are confidently identified, and developing improved methods is an active area of research.

The task of identification extends to inferring peptides and proteins in the sample from the identified MS/MS spectra. This is challenging due to the “many-to-many” mapping of peptides to proteins, and of MS/MS spectra to peptides. Inference must enable parsimonious results, while maintaining the sensitivity and characterizing the confidence in the identifications. The problem of protein inference is not entirely solved. For example, arguments exist in favor [47] and against [48] reporting single-peptide protein identifications, and in favor [49] and against [50] the exclusive use of protease-specific peptides.

A typical experiment generates hundreds of thousands of MS/MS spectra, and open-source and commercial pipelines such as the Trans-Proteomic Pipeline [51] streamline spectral handling and interpretation through common infrastructure.

### Quantification of Spectral Features

The next step in quantitative label-free LC-MS/MS experiments is to locate and quantify MS peaks, annotate them with peptide and sequence identities, and establish the correspondence of peaks between runs [52]. Label-based workflows with MS quantification (e.g., SILAC) search for pairs of peaks with known mass shifts that correspond to a same peptide. Workflows with MS/MS quantification (e.g., iTRAQ) locate and quantify reporter MS/MS fragments. All these tasks can be made difficult by irregular, overlapped, and missing peaks, chromatographic variations between runs, and incomplete and incorrect identifications. As a result, only a subset of the identified proteins is typically quantified [53]. A variety of signal processing software tools are reviewed in [54], and the representative ones are OpenMS [55] for label-based quantification and MaxQuant [56] for quantification with SILAC.

Targeted SRM experiments sidestep the need for identifying and aligning peaks, and signal processing focuses on peak detection, quantification, and annotation. However, difficulties can arise with overlapped or suppressed signals or incorrectly calibrated transitions, and computational methods can help filter out poor quality transitions [57], [58]. Pipelines such as Skyline [59], [60] and ATAQS [61] streamline these tasks.

Frequently, sample handling induces differences in the quantitative signals between runs, and global between-run normalization is necessary to distinguish true biological changes from these artifacts. Two common approaches to global normalization are sample-based and control-based. Sample-based normalization, e.g., quantile normalization or normalization based on the total ion current, makes the best use of the data, but assumes that the majority of features do not change in abundance [62]. Control-based normalization in preferred in experiments with few measurements or many biological changes.

### Finding Differentially Abundant Proteins

Typical statistical goals of quantitative proteomics are *protein quantification*, i.e., estimation of protein concentration in a sample on a relative or absolute scale, and *class comparison*, i.e., determination of proteins that change in average abundance between conditions. To achieve this, it is often necessary to summarize the quantitative information across all the features that pertain to a protein. One such approach is spectral counting [63], which is based on the insight that in global LC-MS/MS peaks from abundant proteins are more frequently selected for fragmentation, and uses the number of identified MS/MS spectra as a proxy for the abundance. The approach involves minimal signal processing; however, it requires specialized statistical modeling, is limited to finding large changes among abundant proteins, and is most successful with mixtures of low complexity, e.g., for determination of protein complexes [64].

Alternative approaches are based on summarizing signals from quantified spectral peaks. With other technologies such as gene expression microarrays, similar summarization is performed by some form of averaging, e.g., with Robust Multiarray Averaging (RMA) [65]. Unfortunately, averaging fails to produce accurate results in mass spectrometry–based proteomics. Length, charge, and other chemical properties of peptides greatly affect the quality of the signals, and averaging obscures these difference in information content.

A more successful summarization requires probabilistic modeling, which represents all features of a protein and characterizes their variation. A diverse range of such models has been proposed, and there is no single generally accepted procedure. The models differ in using raw or log-transformed intensities, comparing groups in terms of ratios or differences, and using general-purpose [66] or specialized [67] classes of statistical models. Important aspects are accurate representation of the experimental design and of within-run groupings of peaks in label-based workflows, treatment of missing data (e.g., using specialized [68] or general-purpose [69], [70] techniques), incorporating confidence in feature identifications [71], expanding the scope of conclusions to the underlying populations or restricting it to the selected samples [66], and controlling the FDR in the list of differentially abundant proteins. In some cases, e.g., in samples enriched in post-translational modifications, changes in peak intensities can be due to both differential abundance and differential modifications. Comparisons at the feature level are then more appropriate; however, they should be adjusted for the overall changes in protein abundance [72].

Given the diversity of experimental designs and analysis steps, all these tasks can rarely be performed in a fully automated fashion, and consultations with statisticians are highly recommended.

### Downstream Analysis

The high-throughput nature of proteomic data is similar to that of gene expression microarrays, and many downstream analysis methods can also be applied in proteomics [73]. In particular, all analyses benefit from data visualization [74]. Unsupervised *class discovery* helps find functionally related proteins, or biological samples homogeneous with respect to the quantitative protein profiles. Supervised *class prediction*, e.g., prediction of the disease status of a patient based on his or her protein abundance [75], and its thorough validation [76], are the required steps for discovery of biomarkers of disease.

*Enrichment analysis* tests whether pre-specified sets of proteins, e.g., those sharing a function, change in abundance more systematically than as expected by chance. This is referred to as *pathway analysis* when the protein set forms a pathway. The analysis investigates hypotheses that are more directly relevant to the biological function, and can help detect small but consistent changes in abundance within the set. Many enrichment analysis methods exist and are systematically reviewed in [77], [78], and representative examples are the hypergeometric (equivalently, Fisher's exact) test and Gene Set Enrichment Analysis (GSEA) [79]. A particular challenge in proteomics is to map the protein identitifiers to gene-centric knowledge bases. The tools for this task are reviewed in [80], and a representative one is DAVID [81].

A frequently asked question is the correlation between the expression of protein-coding genes and the abundances of the corresponding proteins [82]–[84]. Many studies reported that in bacteria and uni-cellular eukaryotes, proteins and mRNA exhibit moderate correlation in a steady state (Pearson correlation of the order of 0.4), but it improves to the order of 0.6–0.7 for proteins that are directly affected by a relevant condition or a stress [2]. An even lower correlation has been historically reported for multi-cellular eukaryotes; however, technological improvements now also point to a steady state correlation in human samples of the order of 0.4 [85].

The moderate correlation of transcript and protein abundance indicates a major role of post-translational regulation in the activity of the cell. Therefore, the best functional insight can be obtained by combining measurements across technologies, and searching for broader groups of genes, proteins, and metabolites forming regulatory relationships [86], [87]. Such integrative studies are increasingly appearing [88], [89]. They remain challenging, however, due to the complexity of the underlying processes, incomplete sampling of the proteome, uncertainty in protein identities and difficulties of resolving multiple proteomic, genomic, and technological identifiers across platforms. New specialized methods and algorithms are needed to address these challenges.

## Outlook

Despite the challenges, mass spectrometry–based proteomics continues to bring high promise for basic science and clinical research [90]. Several studies recently demonstrated that with appropriate care and training, it is now possible to accurately and reproducibly identify and quantify proteins across laboratories and instrument platforms [91]–[93]. In shotgun proteomics, most repeatable peptide identifications corresponded to enzyme-specific cleavage sites, intense MS peaks, and proteins that generated many distinct peptides. Targeted quantification could reproducibly detect low µg/ml protein concentrations in unfractionated plasma.

To date, only 65% of all predicted human proteins have been reliably observed by mass spectrometry [90]. Therefore, future experimental developments will focus on improving the sensitivity, reproducibility, and comprehensiveness of protein identifications, and the sensitivity and accuracy of quantification. All studies consistently emphasize the key role of computation [94]. Future computational efforts will involve the development of proteome-centric knowledge bases such as neXtProt (http://www.nextprot.org/), repositories of experimental data, and the development of methods for optimal experimental design and data interpretation. Venues such as RECOMB Satellite Conference on Computational Proteomics [95] aim at closing the communication gap between biologists, chemists, and statisticians, and enable integrative and collaborative research.

## References

[1] Beck M, Claassen M, Aebersold R (2011). Comprehensive proteomics.. Curr Opin Biotechnol.

[2] de Godoy LMF, Olsen JV, Cox J, Nielsen ML, Hubner NC (2008). Comprehensive mass-spectrometry-based proteome quantification of haploid versus diploid yeast.. Nature.

[3] Olsen JV, Blagoev B, Gnad F, Macek B, Kumar C (2006). Global, in vivo, and site-specific phosphorylation dynamics in signaling networks.. Cell.

[4] Gavin AC, Bösche M, Krause R, Grandi P, Marzioch M (2002). Functional or ganization of the yeast proteome by systematic analysis of protein complexes.. Nature.

[5] Cox J, Mann M (2011). Quantitative, high-resolution proteomics for data-driven systems biology.. Annu Rev Biochem.

[6] Gstaiger M, Aebersold R (2009). Applying mass spectrometry-based proteomics to genetics, genomics and network biology.. Nat Rev Genet.

[7] Castellana N, Bafna V (2010). Proteogenomics to discover the full coding content of genomes: A computational perspective.. J Proteomics.

[8] Ansong C, Purvine S, Adkins J, Lipton M, Smith R (2008). Proteogenomics: needs and roles to be filled by proteomics in genome annotation.. Brief Funct Genomic Proteomic.

[9] Hanash S, Taguchi A (2010). The grand challenge to decipher the cancer proteome.. Nat Rev Cancer.

[10] Uhlen M, Ponten F (2005). Antibody-based proteomics for human tissue profiling.. Mol Cell Proteomics.

[11] Anderson NL, Anderson NG (2002). The human plasma proteome: history, character, and diagnostic prospects.. Mol Cell Proteomics.

[12] Ahrens CH, Brunner E, Qeli E, Basler K, Aebersold R (2010). Generating and navigating proteome maps using mass spectrometry.. Nat Rev Mol Cell Biol.

[13] Corzett TH, Fodor IK, Choi MW, Walsworth VL, Turteltaub KW (2010). Statistical analysis of variation in the human plasma proteome.. J Biomed Biotechno1.

[14] Oberg AL, Vitek O (2009). Statistical design of quantitative mass spectrometry-based proteomic experiments.. J Proteome Res.

[15] Valledor L, Jorrín J (2010). Back to the basics: maximizing the information obtained by quantitative two dimensional gel electrophoresis analyses by an appropriate experimental design and statistical analyses.. J Proteomics.

[16] Ransohoff DF (2005). Bias as a threat to the validity of cancer molecular-marker research.. Nat Rev Cancer.

[17] Hu J, Coombes KR, Morris JS, Baggerly KA (2005). The importance of experimental design in proteomic mass spectrometry experiments: some cautionary tales.. Brief Funct Genomic Proteomic.

[18] Mallick P, Kuster B (2010). Proteomics: a pragmatic perspective.. Nat Biotechnol.

[19] Walther TC, Mann M (2010). Mass spectrometry-based proteomics in cell biology.. J Cell Biol.

[20] Domon B, Aebersold R (2010). Options and considerations when selecting a quantitative proteomics strategy.. Nat Biotechnol.

[21] Duncan MW, Aebersold R, Caprioli RM (2010). The pros and cons of peptide-centric proteomics.. Nat Biotechnol.

[22] Mann M, Michalski A, Cox J (2011). More than 100,000 detectable peptide species elute in single shotgun proteomics runs but the majority is inaccessible to data dependent LC MS/MS.. J Proteome Res.

[23] Ong SE, Mann M (2006). A practical recipe for stable isotope labeling by amino acids in cell culture (SILAC).. Nat Biotechnol.

[24] Ross PL, Huang YN, Marchese JN, Williamson B, Parker K (2004). Multiplexed protein quantitation in saccharomyces cerevisiae using amine-reactive isobaric tagging reagents.. Mol Cell Proteomics.

[25] Thompson A, Schäfer J, Kuhn K, Kienle S, Schwarz J (2003). Tandem mass tags: a novel quantification strategy for comparative analysis of complex protein mixtures by MS/MS.. Anal Chem.

[26] Geiger T, Wisniewski JR, Cox J, Zanivan S, Kruger M (2011). Use of stable isotope labeling by amino acids in cell culture as a spike-in standard in quantitative proteomics.. Nat Protoc.

[27] Rifai N, Gillette MA, Carr SA (2006). Protein biomarker discovery and validation: the long and uncertain path to clinical utility.. Nat Biotechnol.

[28] Yocum AK, Chinnaiyan AM (2009). Current affairs in quantitative targeted proteomics: Multiple reaction monitoring-mass spectrometry.. Brief Funct Genomic Proteomic.

[29] Kitteringham NR, Jenkins RE, Lane CS, Elliott VL, Park BK (2009). Multiple reaction monitoring for quantitative biomarker analysis in proteomics and metabolomics.. J Chromatogr B.

[30] Pan S, Aebersold R, Chen R, Rush J, Goodlett DR (2009). Mass spectrometry based targeted protein quantification: methods and applications.. J Proteome Res.

[31] Lange V, Picotti P, Domon B, Aebersold R (2008). Selected reaction monitoring for quantitative proteomics: a tutorial.. Mol Sys Biol.

[32] Picotti P, Bodenmiller B, Mueller LN, Domon B, Aebersold R (2009). Full dynamic range proteome analysis of S. cerevisiae by targeted proteomics.. Cell.

[33] Picotti P, Rinner O, Stallmach R, Dautel F, Farrah T (2010). High-throughput generation of selected reaction-monitoring assays for proteins and proteomes.. Nat Methods.

[34] Bertsch A, Jung S, Zerck A, Pfeifer N, Nahnsen S (2010). Optimal *de novo* design of MRM experiments for rapid assay development in targeted proteomics.. J Proteome Res.

[35] Granholm V, Käll L (2011). Quality assessments of peptide?spectrum matches in shotgun proteomics.. Proteomics.

[36] Nesvizhskii AI (2010). A survey of computational methods and error rate estimation procedures for peptide and protein identification in shotgun proteomics.. J Proteomics.

[37] Nesvizhskii AI, Vitek O, Aebersold R (2007). Analysis and validation of proteomic data generated by tandem mass spectrometry.. Nat Methods.

[38] Lam H, Aebersold R (2011). Building and searching tandem mass (MS/MS) spectral libraries for peptide identification in proteomics.. Methods.

[39] Jeong K, Kim S, Bandeira N, Pevzner PA (2011). Gapped spectral dictionaries and their applications for database searches of tandem mass spectra.. Mol Cell Proteomics.

[40] Dasari S, Chambers M, Slebos R, Zimmerman L, Ham A (2010). TagRecon: high-throughput mutation identification through sequence tagging.. J Proteome Res.

[41] Venable JD, Yates JR (2004). Impact of ion trap tandem mass spectra variability on the identification of peptides.. Anal Chem.

[42] Carr S, Aebersold R, Baldwin M, Burlingame A, Clauser K (2004). The need for guidelines in publication of peptide and protein identification data.. Mol Cell Proteomics.

[43] Käll L, Storey J, MacCoss M, Noble W (2008). Assigning significance to peptides identified by tandem mass spectrometry using decoy databases.. J Proteome Res.

[44] H C, I NA (2008). False discovery rates and related statistical concepts in mass spectrometry-based proteomics.. J Proteome Res.

[45] Keller A, Nesvizhskii AI, Kolker E, Aebersold R (2002). Empirical statistical model to estimate the accuracy of peptide identifications made by MS/MS and database search.. Anal Chem.

[46] Moore R, Young M, Lee T (2002). Qscore: an algorithm for evaluating SEQUEST database search results.. J Am Soc Mass Spectrom.

[47] Gupta N, Pevzner PA (2009). False discovery rates of protein identifications: a strike against the two-peptide rule.. J Proteome Res.

[48] Reiter L, Claassen M, Schrimpf S, Jovanovic M, Schmidt A (2009). Protein identification false discovery rates for very large proteomics data sets generated by tandem mass spectrometry.. Mol Cell Proteomics.

[49] Olsen JV, Ong SE, Mann M (2004). Trypsin cleaves exclusively C-terminal to arginine and lysine residues.. Mol Cell Proteomics.

[50] Gupta N, Hixson KK, Culley DE, Smith RD, Pevzner PA (2010). Analyzing protease specificity and detecting in vivo proteolytic events using tandem mass spectrometry.. Proteomics.

[51] Deutsch EW, Mendoza L, Shteynberg D, Farrah T, Lam H (2010). A guided tour of the Trans-Proteomic Pipeline.. Proteomics.

[52] America AHP, Cordewener JHG (2008). Comparative LC-MS: a landscape of peaks and valleys.. Proteomics.

[53] Schulze WX, Usadel B (2010). Quantitation in mass-spectrometry-based proteomics.. Annu Rev Plant Biol.

[54] Mueller LN, Brusniak MY, Mani DR, Aebersold R (2008). An assessment of software solutions for the analysis of mass spectrometry based quantitative proteomics data.. J Proteome Res.

[55] Sturm M, Bertsch A, Gröpl C, Hildebrandt A, Hussong R (2008). OpenMS – An open-source software framework for mass spectrometry.. BMC Bioinformatics.

[56] Cox J, Mann M (2008). MaxQuant enables high peptide identification rates, individualized p.p.b.-range mass accuracies and proteome-wide protein quantification.. Nat Biotechnol.

[57] Abbatiello S, Mani DR, Keshishian H, Carr S (2010). Automated detection of inaccurate and imprecise transitions in peptide quantification by multiple reaction monitoring mass spectrometry.. Clin Chem.

[58] Reiter L, Rinner O, Picotti P, Hüttenhain R, Beck M (2011). mProphet: automated data processing and statistical validation for large-scale SRM experiments.. Nat Methods.

[59] MacLean B, Tomazela D, Shulman N, Chambers M, Finney G (2010). Skyline: an open source document editor for creating and analyzing targeted proteomics experiments.. Bioinformatics.

[60] Cham Mead JA, Bianco L, Bessant C (2010). Free computational resources for designing selected reaction monitoring transitions.. Proteomics.

[61] Brusniak MYK, Kwok ST, Christiansen M, Campbell D, Reiter L (2011). ATAQS: a computational software tool for high throughput transition optimization and validation for selected reaction monitoring mass spectrometry.. BMC Bioinformatics.

[62] Callister SJ, Barry RC, Adkins JN, Johnson ET, Qian WJ (2006). Normalization approaches for removing systematic biases associated with mass spectrometry and label-free proteomics.. J Proteome Res.

[63] Lundgren DH, Hwang S, Wu L, Han DK (2010). Role of spectral counting in quantitative proteomics.. Expert Rev Proteomics.

[64] Choi H, Larsen B, Lin ZY, Breitkreutz A, Mellacheruvu D (2010). SAINT: probabilistic scoring of affinity purification-mass spectrometry data.. Nat Methods.

[65] Irizarry RA, Hobbs B, Collin F, Beazer-Barclay YD, Antonellis KJ (2003). Exploration, normalization, and summaries of high density oligonucleotide array probe level data.. Biostatistics.

[66] Clough T, Key M, Ott I, Ragg S, Schadow G (2009). Protein quantification in label-free LC-MS experiments.. J Proteome Res.

[67] Griffin NM, Yu J, Long F, Oh P, Shore S (2010). Label-free, normalized quantification of complex mass spectrometry data for proteomic analysis.. Nat Biotechnol.

[68] Karpievitch Y, Stanley J, Taverner T, Huang J, Adkins JN (2009). A statistical framework for protein quantitation in bottom-up MS-based proteomics.. Bioinformatics.

[69] Liew AW, Law NF, Yan H (2010). Missing value imputation for gene expression data: computational techniques to recover missing data from available information.. Brief Bioinform.

[70] Aittokallio T (2010). Dealing with missing values in large-scale studies: microarray data imputation and beyond.. Brief Bioinform.

[71] Li YF, Arnold RJ, Tang H, Radivojac P (2010). The importance of peptide detectability for protein identification, quantification, and experiment design in MS/MS proteomics.. J Proteome Res.

[72] Wu R, Dephoure N, Haas W, Huttlin EL, Zhai B (2011). Correct interpretation of comprehensive phosphorylation dynamics requires normalization by protein expression changes.. Mol Cell Proteomics.

[73] Kumar C, Mann M (2009). Bioinformatics analysis of mass spectrometry-based proteomics data sets.. FEBS Letters.

[74] Gehlenborg N, O'Donoghue SI, Baliga NS, Goesmann A, Hibbs MA (2010). Visualization of omics data for systems biology.. Nat Methods.

[75] Clarke R, Ressom HW, Wang A, Xuan J, Liu MC (2008). The properties of high-dimensional data spaces: implications for exploring gene and protein expression data.. Nat Rev Cancer.

[76] Boulesteix AL, Sauerbrei W (2011). Added predictive value of high-throughput molecular data to clinical data and its validation.. Brief Bioinform.

[77] Emmert-Streib F, Glazko GV (2011). Pathway analysis of expression data: deciphering functional building bocks of complex diseases.. PLoS Comp Biol.

[78] Ackermann M, Strimmer K (2009). A general modular framework for gene set enrichment analysis.. BMC Bioinformatics.

[79] Subramanian A, Tamayo P, Mootha VK, Mukherjee S, Ebert BL (2005). Gene set enrichment analysis: a knowledge-based approach for interpreting genome-wide expression profiles.. Proc Natl Acad Sci U S A.

[80] Huang D, Sherman BT, Lempicki R (2009). Bioinformatics enrichment tools: paths toward the comprehensive functional analysis of large gene lists.. Nucleic Acids Res.

[81] Huang DW, Sherman BT, Lempicki RA (2009). Systematic and integrative analysis of large gene lists using david bioinformatics resources.. Nat Protocols.

[82] de Sousa Abreu R, Penalva LO, Marcotte EM, Vogel C (2009). Global signatures of protein and mRNA expression levels.. Mol BioSystems.

[83] Maier T, Guell M, Serrano L (2009). Correlation of mRNA and protein in complex biological samples.. FEBS Lett.

[84] Nie L, Wu G, Culley DE, Scholten JCM, Zhang W (2007). Integrative analysis of transcriptomic and proteomic data: challenges, solutions and applications.. Crit Rev Biotechnol.

[85] Schwanhäusser B, Busse D, Li N, Dittmar G, Schuchhardt J (2011). Global quantification of mammalian gene expression control.. Nature.

[86] Joyce AR, Palsson BØ (2006). The model organism as a system: integrating ‘omics’ data sets.. Nat Rev Mol Cell Biol.

[87] Sharan R, Ulitsky I, Shamir R (2007). Network-based prediction of protein function.. Mol Syst Biol.

[88] Nibbe RK, Koyuturk M, Chance MR (2010). An integrative -omics approach to identify functional sub-networks in human colorectal cancer.. PLoS Comp Biol.

[89] Huang SS, Fraenkel E (2010). Integration of proteomic, transcriptional, and interactome data reveals hidden signaling components.. Sci Signal.

[90] Nilsson T, Mann M, Aebersold R, Yates JR, Bairoch A (2010). Mass spectrometry in high-throughput proteomics: ready for the big time.. Nat Methods.

[91] Tabb DL, Vega-Montoto L, Rudnick P, Variyath A, Ham A (2010). Repeatability and reproducibility in proteomic identifications by liquid chromatography-tandem mass spectrometry.. J Proteome Res.

[92] Bell A, Deutsch E, Au C, Kearney R, Beavis R (2009). A HUPO test sample study reveals common problems in mass spectrometry–based proteomics.. Nat Methods.

[93] Addona TA, Abbatiello SE, Schilling B, Skates SJ, Mani DR (2009). Multi-site assessment of the precision and reproducibility of multiple reaction monitoring-based measurements of proteins in plasma.. Nat Biotechnol.

[94] Aebersold R (2009). A stress test for mass spectrometry-based proteomics.. Nat Methods.

[95] Bandeira N, Nesvizhskii A, McIntosh M (2011). Advancing next-generation proteomics through computational research.. J Proteome Res.

